# Interactions of a Dopamine D_1_ Receptor Agonist with Glutamate NMDA Receptor Antagonists on the Volitional Consumption of Ethanol by the mHEP Rat

**DOI:** 10.3390/ph6040469

**Published:** 2013-03-26

**Authors:** Brian A. McMillen, Courtney L. Lommatzsch, Michael J. Sayonh, Helen L. Williams

**Affiliations:** Department of Pharmacology & Toxicology, Brody School of Medicine at East Carolina University, Greenville, NC 27834, USA; E-Mails: clommatzsch@yahoo.com (C.L.L.); mjsayonh@hotmail.com (M.J.S.); williamshe@ecu.edu (H.L.W.)

**Keywords:** glutamate receptors, consumatory behaviors, DARPP-32

## Abstract

Stimulation of the dopamine D1 receptor is reported to cause the phosphorylation of DARPP-32 at the thre34 position and activates the protein. If intracellular Ca^2+^ is increased, such as after activation of the glutamate NMDA receptor, calcineurin activity increases and the phosphates will be removed. This balance of phosphorylation control suggests that a D_1_ receptor agonist and a NMDA glutamate receptor antagonist should have additive or synergistic actions to increase activated DARPP-32 and consequent behavioral effects. This hypothesis was tested in a volitional consumption of ethanol model: the selectively bred Myers’ high ethanol preferring (mHEP) rat. A 3-day baseline period was followed by 3-days of twice daily injections of drug(s) or vehicle(s) and then a 3-day post-treatment period. Vehicle, the D_1_ agonist SKF 38393, the non-competitive NMDA receptor antagonist memantine, or their combination were injected 2 h before and after lights out. The combination of 5.0 mg/kg SKF 38393 with either 3.0 or 10 mg/kg memantine did not produce an additive or synergistic effect. For example, 5.0 mg/kg SKF reduced consumption of ethanol by 27.3% and 10 mg/kg memantine by 39.8%. When combined, consumption declined by 48.2% and the proportion of ethanol solution to total fluids consumed declined by 17%. However, the consumption of food also declined by 36.6%. The latter result indicates that this dose combination had a non-specific effect. The combination of SKF 38393 with (+)-MK-801, another NMDA receptor antagonist, also failed to show an additive effect. The lack of additivity and specificity suggests that the hypothesis may not be correct for this *in vivo* model. The interaction of these different receptor systems with intraneuronal signaling and behaviors needs to be studied further.

## 1. Introduction

Ethanol interacts with the functioning of many different neurotransmitter systems. These interactions include increased dopaminergic impulse flow likely through an opiate receptor mediated system [1], potentiation of gamma-aminobutyric acid (GABA) at the GABA_A_ receptor, inhibition of excitatory glutamate (NMDA) receptors [2,3], release of angiotensin II [4] and an agonist-like interaction with adenosine receptors [5,6]. The search for drugs that will reduce the desire to consume alcohol similarly involves modification of the function of these neurotransmitter-receptor systems. One such example is the use of opiate receptor antagonists such as naltrexone to reduce consumption of ethanol in animal models [7,8] and in humans [9,10]. Drugs that either enhance or diminish serotonergic function have been tried in both animal models and clinical trials with varying degrees of success [11,12,13] that may be due to differences in response by different sub-types of alcohol-dependent subjects [14].

In addition to the acceptor site for glutamate itself, the glutamate NMDA receptor has several different binding sites that can be targeted to modify the functioning of the receptor. Drugs that target this receptor to reduce glutamate activation will reduce the volitional consumption of ethanol by selectively bred high ethanol preferring rats. Thus, a drug that directly inhibits the binding of glutamate to this ligand-operated channel, LY 274614 (3*SR*,4a*RS*,6*SR*,8a*RS*-6-[phosphonomethyl]-decahydroisoquinoline-3-carboxylic acid), drugs that inhibit the binding of glycine to an accessory site on the receptor, (+)-HA-966 (3-amino-1-hydroxy-2-pyrrolidone) and ACPC (1-aminocyclo-propane-1-carboxylic acid), a drug that binds to the “PCP-site” to potently and non-competitively inhibit, dizolcipine (MK-801), or a drug that blocks the opened channel, memantine, all will decrease volitional consumption at doses with insignificant or small effects on food intake and motor performance [15,16]. In humans, pre-treatment of moderate drinkers with memantine reduced the craving for alcohol prior to their receiving alcohol, but not afterwards [17,18]. However, memantine did not alter drinking patterns of moderate drinkers (6–8 drinks per day) who met DSM-IV criteria for alcohol-dependence [19]. The subjects in this latter study did not appear to meet the description for type 2 early onset heavy drinkers [20,21].

The glutamate NMDA receptor is a ligand-operated Ca^2+^ channel. When the neuronal membrane becomes less polarized, the activation of the NMDA receptor by glutamate will allow Ca^2+^ to flow into the cell until repolarization allows Mg^2+^ move back into the channel. The influx of Ca^2+^ will contribute to an excitatory postsynaptic potential that makes the neuron more likely to discharge and the Ca^2+^ will interact with intracellular proteins. One such calcium-activated enzyme is Ca^2+^-/calmodulin-dependent phosphatase, calcineurin. In dopaminergic-responsive neurons, this enzyme will remove a phosphate from DARPP-32 (dopamine and cAMP-regulated phosphoprotein of M_r_ 32 kDa) at Thr34 and decrease its activity. The activation of dopamine D_1_ receptors increases the production of cAMP, in turn activating PKA, which puts a phosphate on DARPP-32 at Thr34 and activate the enzyme. NMDA agonists will decrease the activation of DARPP-32 due to Ca^2+^ activation of calcineurin [22]. This raises the interesting hypothesis that if memantine could reduce the activity of calcineurin by decreasing the intracellular levels of Ca^2+^, DARPP-32 would remain activated longer, and there may be an additive or possibly a synergistic action between a D_1_ agonist and this inhibitor of the NMDA receptor. The following experiments test this hypothesis through the use of the selectively bred Myers’ high ethanol preferring (mHEP) rat [23] that voluntarily consumes large amounts of ethanol solutions and injections of varying doses of a D_1_ partial agonist, SKF 38393 [(±)-1-phenyl-2,3,4,5-tetrahydro-(1*H*)-3-benzazepine-7,8-diol] and either memantine or (+)MK-801, both non-competitive inhibitors of the glutamate NMDA receptor [24].

## 2. Experimental

*Animals*: The Myers mHEP rats were selectively bred and maintained in the vivarium at East Carolina University under a 12-hour lights on/off schedule. The progenitors for this line were three alcohol preferring (P) rats purchased from Indiana University by R. D. Myers and bred with female Sprague-Dawley rats [23] and are known to have low concentrations of serotonin in several brain areas [25]. All procedures were approved by the Institutional Animal Care and Use Committee and were in compliance with the *NIH Guide for the Care and Use of Laboratory Animals*.

*Procedures*: Male rats from the F31–F34 generations were moved to cages with three drinking tubes mounted on the front, one contained tap water, one contained ethanol in tap water and one was left empty, and the positions rotated in a semi-random order daily. Rats were first subjected to a 10-day step-up procedure [23] with the ethanol concentration increased each day from 3% to 30%. Body weight and the consumption of food and fluids were measured daily. The concentration of ethanol for each rat that resulted in the greatest amount of ethanol consumed with a proportion of ethanol solution to total fluids consumed closest to 50% was then used as that rat’s preferred concentration for the remaining experiments.

In order to test the effects of drug combinations on consumatory behavior: a group of rats were divided into two sub-groups in order to make drug and vehicle injections in a counter-balanced design. For each treatment cycle there would be a 3-day pre-treatment period, followed by 3-days of injections made at 2 hours before and 2 hours after lights out and then a 3-day post-treatment period. After drinking returned to baseline levels, each rat was put through the cycle again. Injections consisted of vehicles for both SKF 38393 and memantine (Veh/Veh); the vehicle for SKF 38393 and a dose of memantine injected i.p. (Veh/Mem); a dose of SKF 38393 injected s.c. and the vehicle for memantine (SKF/Veh); or doses of both drugs (SKF/Mem). Each rat served as its own control at one dose combination of SKF 38393 and memantine. An additional group of rats was used with SKF 38393 and (+)-MK 801 as the non-competitive NMDA receptor antagonist. Consumption was averaged for each of the three-day periods, then analyzed by repeated measures ANOVA and Tukey’s HSD test [26] with the aid of PRISM software.

*Drugs*: SKF 38393, [(±)-1-phenyl-2,3,4,5-tetrahydro-(1*H*)-3-benzazepine-7,8-diol)・HCl], memantine hydrochloride and (+)-MK-801 hydrogen maleate were purchased from Sigma-Aldrich (St. Louis, MO, USA). Drugs were dissolved in distilled water and doses were calculated as the free base. Saline was used as the vehicle control for all drugs.

## 3. Results

Table 1 shows the results from the interaction of 2.5 mg/kg s.c. SKF 38393 and 1.0 mg/kg i.p. memantine on the consumatory behavior of the mHEP rat. These doses were chosen as being minimal effective doses for a decrease in the consumption of ethanol [16,27]. As can be seen, there were small decreases in consumption with either drug alone, but no additivity when the drugs were combined, only a 17.3% decrease in the amount consumed compared to the pre-treatment baseline. In addition, proportion of ethanol to total fluid intake (or preference) did not change and there was a small decrease in food consumption. Body weight continued to increase in all four groups (data not shown).

Table 1Effects of 2.5 mg/kg SKF-38393, 1.0 mg/kg memantine, or in combination b.i.d. for three days on daily ethanol consumption, proportion of fluids consumed and food intake.

**Table:** A. Consumption--**g ethanol/kg/day**

	Pre	During	Post
Veh/Veh	5.78 ± 0.62	5.83 ± 0.63	6.33 ± 0.56
Veh/Mem	6.34 ± 0.65	4.85 ± 0.67 *	5.63 ± 0.81
Veh/SKF	6.99 ± 0.51	5.13 ± 0.61 *	5.73 ± 0.72
Mem/SKF	5.84 ± 0.64	4.83 ± 0.51 *	6.23 ± 0.64

**Table:** B. Proportion--**mL ethanol/mL total fluids**

	Pre	During	Post
Veh/Veh	0.59 ± 0.04	0.60 ± 0.05	0.58 ± 0.04
Veh/Mem	0.56 ± 0.04	0.61 ± 0.06	0.58 ± 0.05
Veh/SKF	0.64 ± 0.03	0.65 ± 0.04	0.64 ± 0.06
Mem/SKF	0.58 ± 0.04	0.57 ± 0.05	0.57 ± 0.05

**Table:** C. Consumption of Food--**g food/day**

	Pre	During	Post
Veh/Veh	20.2 ± 1.1	20.2 ± 1.1	20.9 ± 1.4
Veh/Mem	21.8 ± 1.8	20.1 ± 1.2	20.3 ± 0.9
Veh/SKF	21.6 ± 1.2	19.3 ± 1.1 *	20.2 ± 1.3
Mem/SKF	20.5 ± 1.3	18.4 ± 0.8 *	20.8 ± 1.1

Values are Mean ± Standard Error; * Different from pre-treatment baseline, *p* < 0.05 (Tukey’s HSD test), N = 9.

At the dose of 5.0 mg/kg s.c. SKF-38393 there occurred a significant decrease from the pre-treatment period in the consumption of ethanol by 42.8% and the proportion declined by 31.1% (Table 2). Food intake did not change in contrast to the first experiment. Memantine at a dose of 3.0 mg/kg i.p. produced a decrease in consumption of 19.6%, but did not cause a significant decline in the preference. When the two drugs were combined there was a 37.7% decrease in the consumption of ethanol along with a significant decrease in the proportion, but neither value was different from SKF-38393 alone. In this experiment, there were no significant changes in food intake and the rats continued to add body weight throughout the injection sequences.

Table 2Effects of 5.0 mg/kg SKF-38393, 3.0 mg/kg memantine, or in combination b.i.d. for three days on daily ethanol consumption, proportion of fluids consumed and food intake.

**Table:** A. Consumption--**g ethanol/kg/day**

	Pre	During	Post
Veh/Veh	7.91 ± 0.49	7.40 ± 0.36	7.99 ± 0.29
Veh/Mem	7.70 ± 0.38	6.19 ± 0.57 *	7.13 ± 0.48
SKF/Veh	7.63 ± 0.52	4.36 ± 0.51 *	7.82 ± 0.65
Mem/SKF	7.38 ± 0.51	4.60 ± 0.40 *	7.50 ± 0.61

**Table:** B. Proportion--**mL ethanol/mL total fluids**

	Pre	During	Post
Veh/Veh	0.63 ± 0.03	0.67 ± 0.03	0.66 ± 0.02
Veh/Mem	0.64 ± 0.03	0.59 ± 0.04	0.59 ± 0.03
SKF/Veh	0.61 ± 0.02	0.42 ± 0.04 *	0.59 ± 0.04
Mem/SKF	0.60 ± 0.03	0.50 ± 0.04 *	0.65 ± 0.03

**Table:** C. Consumption of Food--**g food/day**

	Pre	During	Post
Veh/Veh	20.4 ± 0.9	19.5 ± 0.9	19.7 ± 1.2
Veh/Mem	20.0 ± 1.0	20.5 ± 1.1	20.6 ± 1.1
SKF/Veh	21.3 ± 1.0	20.3 ± 0.9	20.9 ± 0.9
Mem/SKF	19.6 ± 1.1	19.4 ± 0.9	20.3 ± 1.0

Values are Mean ± Standard Error; * Different from pre-treatment baseline, *p* < 0.05 (Tukey’s HSD test), N = 11.

Table 3 shows the effects of 5.0 mg/kg s.c. SKF 38393 and 10 mg/kg i.p. memantine, doses previously demonstrated as being effective doses for a decrease in the consumption of ethanol. There were significant decreases in consumption with either drug alone: 27% with SKF and 40% with memantine; but no additivity when the drugs were combined, a 47% decrease in the amount consumed that is similar to memantine alone. However, the proportion of ethanol to total fluid intake (or preference) decreased only with the drug combination, but there was a robust decrease in food consumption with memantine alone (23.1%) or in combination with SKF 38393 (33.3%). This dose of memantine was producing an anti-caloric or other non-specific effect in these animals. Body weight was maintained or increased in all four groups.

In Table 4, a dose of SKF 38393 that has been at threshold for significant effects on drinking was combined with a dose of (+)-MK-801 predicted to also be effective on reducing consumption of ethanol based on experience with the racemic mixture [16]. After the dose of 2.5 mg/kg s.c. SKF-38393 there occurred a significant decrease in the consumption of ethanol by 34% and the proportion declined by 19% compared to the pre-treatment period, both slightly higher than shown in Table 1 with rats from an earlier generation. MK-801 at a dose of 0.1 mg/kg i.p. produced a decrease in consumption of 32% from the pre-treatment period, but did not cause a significant decline in the preference. When the two drugs were combined there was a 21% decrease in the consumption of ethanol along with a significant decrease in the proportion, but neither value was different from SKF-38393 alone. In this experiment, there were small changes in food intake and the rats continued to add body weight throughout the injection sequences.

Table 3Effects of 5.0 mg/kg SKF-38393, 10 mg/kg memantine, or in combination b.i.d. for three days on daily ethanol consumption, proportion of fluids consumed and food intake.

**Table:** A. Consumption--**g ethanol/kg/day**

	Pre	During	Post
Veh/Veh	6.55 ± 0.40	6.30 ± 0.33	6.18 ± 0.35
Veh/Mem	6.23 ± 0.44	3.75 ± 0.54 *^#^	5.87 ± 0.61
SKF/Veh	6.31 ± 0.35	4.59 ± 0.31 *^#^	6.02 ± 0.55
Mem/SKF	6.27 ± 0.52	3.25 ± 0.51 *^#^	6.08 ± 0.56

**Table:** B. Proportion--**mL ethanol/mL total fluids**

	Pre	During	Post
Veh/Veh	0.72 ± 0.03	0.74 ± 0.03	0.66 ± 0.03
Veh/Mem	0.66 ± 0.03	0.64 ± 0.05	0.65 ± 0.03
SKF/Veh	0.71 ± 0.02	0.63 ± 0.03	0.69 ± 0.04
Mem/SKF	0.65 ± 0.02	0.54 ± 0.05 *^#^	0.70 ± 0.03

**Table:** C. Consumption of Food--**g food/day**

	Pre	During	Post
Veh/Veh	21.5 ± 0.9	19.5 ± 0.9 *	20.4 ± 0.8
Veh/Mem	21.0 ± 0.7	15.0 ± 1.1 *^#^	19.1 ± 0.6
SKF/Veh	21.3 ± 0.9	19.7 ± 0.7 *	21.4 ± 1.0
Mem/SKF	20.5 ± 0.5	13.0 ± 0.9 *^#^	20.7 ± 0.9

Values are Mean ± Standard Error; *Different from pre-treatment baseline, # different from Veh/Veh, *p* < 0.05 (Tukey’s HSD test), N = 9.

## 4. Discussion

Ethanol has the effect of causing the dopaminergic neurons in the mid-brain to discharge at increased rates and the effect is mediated through opiate receptors [1] and ethanol increases extracellular concentrations of dopamine [28]. The hypothesis is that metabolism of monoamines by monoamine oxidase provides hydrogen peroxide equivalents for catalase to oxidize ethanol to acetaldehyde which then condenses with monoamines to form morphine-like compounds [29,30]. Stimulation of D_1_ receptors (25 µm SKF 38393) will increase production of cAMP, increase PKA activity, and activate DARPP-32. In turn, the NMDA receptor will become more phosphorylated and less sensitive to inactivation by ethanol [31]. Elimination of expression of either the D_1_ receptor or DARPP-32 in mice reduces ethanol-seeking behaviors [32,33]. In very large doses, amphetamines are neurotoxic to the dopaminergic neurons and these neurons can be protected by the NMDA receptor antagonist, MK-801 [34], a result that suggests important interactions between dopamine and glutamate *in vivo*.

Table 4Effects of 2.5 mg/kg SKF-38393, 0.1 mg/kg (+)-MK-801 or in combination b.i.d. for three days on daily ethanol consumption, proportion of fluids consumed and food intake.

**Table:** A. Consumption--**g ethanol/kg/day**

	Pre	During	Post
Veh/Veh	5.88 ± 0.36	5.51 ± 0.28	5.40 ± 0.22
Veh/Mem	5.80 ± 0.16	3.93 ± 0.25 *^#^	4.42 ± 0.23 *
SKF/Veh	5.78 ± 0.28	3.82 ± 0.40 *^#^	5.82 ± 0.25
Mem/SKF	4.92 ± 0.32	3.89 ± 0.19 *^#^	5.22 ± 0.25

**Table:** B. Proportion--**mL ethanol/mL total fluids**

	Pre	During	Post
Veh/Veh	0.66 ± 0.04	0.65 ± 0.04	0.59 ± 0.04 *
Veh/Mem	0.65 ± 0.03	0.57 ± 0.05 *	0.53 ± 0.03 *
SKF/Veh	0.64 ± 0.03	0.52 ± 0.05 *^#^	0.66 ± 0.04
Mem/SKF	0.57 ± 0.03	0.54 ± 0.03 ^#^	0.62 ± 0.04

**Table:** C. Consumption of Food--**g food/day**

	Pre	During	Post
Veh/Veh	20.6 ± 0.7	20.2 ± 0.7	20.9 ± 0.7
Veh/Mem	21.6 ± 0.9	22.0 ± 1.2	22.4 ± 0.8
SKF/Veh	21.0 ± 0.6	19.6 ± 0.7 *	21.6 ± 0.7
Mem/SKF	22.3 ± 0.9	20.2 ± 0.9 *	20.7 ± 0.8

Values are Mean ± Standard Error; * Different from pre-treatment baseline, ^#^ different from Veh/Veh, *p* < 0.05 (Tukey’s HSD test), N = 10.

Stimulation of the D_1_ receptor will reduce consumption of ethanol in a limited access paradigm [35] and during operant responding for ethanol [36]. Systemic injection of either a D_1_ receptor agonist, SKF 38393, or a D_1_ receptor antagonist, SCH 23390, will reduce volitional consumption of ethanol by the mHEP rat [27]. That both an agonist and an antagonist at the D_1_ receptor reduce drinking suggests that deviation in either direction from normal dopaminergic function reduces the rewarding action of ethanol. SKF 38393 is a partial agonist at the D_1_ receptor and produces different behavioral effects as the dose is increased, but hyperactivity is not one [37]. When used as the stimulus in a drug discrimination paradigm, rats can detect SKF 38393 at the doses used in this study [38] and these doses will enhance locomotion induced by a D_2_ agonist [39]. If ethanol is thought of as an indirect DA agonist through enhanced release of DA, then the use of another agonist to reduce consumption is a standard ploy of addiction treatment.

The hypothesis that a D_1_ receptor agonist and an NMDA receptor antagonist should add or synergize to reduce the consumption of ethanol was based on the report by Nishi and co-workers [22]. A D_1_ receptor agonist will activate DARPP-32 through the cAMP/PKA pathway and a NMDA agonist will counter the effect due to increased intracellular Ca^2+^ and activation of calcineurin. Their work suggests that a NMDA receptor antagonist would either further increase activated DARPP-32 or allow the enzyme to remain in the activated phosphorylated state. Doses of a D_1_ receptor agonist and NMDA antagonist were used that previously were demonstrated to decrease volitional consumption of ethanol with minimal reductions in food intake or locomotor disruption. Contrary to the hypothesis, there was neither an additive nor synergistic interaction when such drugs were combined. Neither memantine nor MK-801 combined with SKF 38393 caused a decrease of the consumption of ethanol than that seen with the D_1 _agonist alone. Table 5 summarizes the percent decrease in consumption from the 3-day vehicle/vehicle treatment period produced by the different drugs and combinations. A dose-related inhibition by both memantine and SKF 38393 are evident, but no additivity or synergy when the drugs are combined.

**Table 5:** Summary of data from the Table 1, Table 2, Table 3, Table 4. Percent decrease in the consumption (g/kg/day) of ethanol during treatment with SKF 38393 and either memantine or (+)-MK-801 compared to the value during vehicle treatment are shown.

	Vehicle	1.0 Mem	3.0 Mem	10 Mem	0.1 MK
Vehicle	--	16.8%	16.4%	40.5%	28.7%
2.5 SKF (Tbl 1) (Tbl 4)	12.0%30.7%	17.3%--	----	----	--29.4%
5.0 SKF (Tbl 2) (Tbl 3)	41.1%37.1%	----	37.8%--	--48.4%	----

One explanation for the failure is that the shift in phosphorylation of DARPP-32 at Thre34 has no influence on the consumption of ethanol. It would be necessary to measure total and threo-34 phospho-DARPP-32 by western blot to determine whether changes in phosphorylation are occurring as predicted from in vitro studies. However, normally ethanol-seeking mice with DARPP-32 knocked out do not seek ethanol [33]. The C57bl mouse consumes ethanol largely for the calories [40], whereas the mHEP rat apparently consumes for a pharmacological effect [23]. It is possible that the signaling through the DARPP-32 phosphoprotein cascade is not related to the rewarding effect of ethanol in the mHEP rat. Also, an increase in intracellular Ca^2+^ will alter the activity of many enzymes in addition to calcineurin and the effects in a DARPP-32 expressing neuron will be different than in a neuron that does not.

Another possible explanation is that memantine is too weak of an inhibitor of the NMDA glutamate receptor and perhaps is insufficient to reduce the calcium influx and activation of calcineurin (PP-2B). However, MK-801 is a more potent inhibitor of this receptor, but had no better effect when combined with SKF 38393. Or, it could be a matter of dosing. The doses of memantine and MK-801 were chosen based on prior work to have minimal motor and food consumatory effects. Large doses of these drugs produce an abnormal locomotor behavior, different from amphetamine-like stimulants (personal observation) and in the case of MK-801 in extreme can produce a ketamine-like dissociative anesthetic state. The goal was to avoid these extreme effects and in order to demonstrate synergy, threshold effective doses should combine to produce a strong effect on consumption. Simply, this was not seen.

Finally, it may be possible that with the *in vivo* situation other sources of calcium are available to activate calcineurin so that de-phosphorylation of DARPP-32 still occurs. That is, the amount of calcium entry associated with the NMDAR is small compared to other sources of calcium within the cell. The hypothesis was based on the results obtained *in vitro* by the application of drugs to striatal tissue slices [22] and may not apply to the whole animal system or the concentrations of memantine and MK-801 from injection do not approach the degree of inhibition obtained in the slice preparations. An alternative hypothesis is simply that these drugs reduce the activation of dopaminergic neurons by glutamate and reduce the amount of dopamine released in reward areas such as the *nucleus accumbens* and medial pre-frontal cortex. More research needs to be done to determine the mechanism of interaction of NMDA receptor drugs with ethanol on dopaminergic activity and dopamine release.
